# Phylogeographic pattern of the plane leaf miner, *Phyllonorycter platani* (STAUDINGER, 1870) (Lepidoptera: Gracillariidae) in Europe

**DOI:** 10.1186/s12862-018-1240-z

**Published:** 2018-09-06

**Authors:** Viktória Tóth, Ferenc Lakatos

**Affiliations:** Institute of Silviculture and Forest Protection, Faculty of Forestry, University of Sopron, Bajcsy-Zsilinszky u. 4, Sopron, H-9400 Hungary

**Keywords:** *Phyllonorycter platani*, Phylogeography, mtDNA, Refuges, Native area, Introgression

## Abstract

**Background:**

The plane leaf miner, *Phyllonorycter platani* is a widely distributed insect species on plane trees and has a well-documented colonisation history in Europe over the last century. However, phylogeographic data of the species are lacking.

**Results:**

We analysed 284 individuals from 38 populations across Europe, Asia, and North America. A 1242 bp fragment of the mitochondrial COI gene and an 893 bp fragment of the 28S rDNA has been Sanger sequenced. Twenty-four haplotypes were detected on the COI gene, and two alleles were identified on the 28S rDNA. We revealed two distinct clades for both markers reflecting the geographic origins, Asia and Europe. The genetic distance between the two main clades is 2.08% on the COI gene and 0.10% on the nuclear DNA.

An overlapping zone of the two clades was found across Eastern Europe and the Anatolian Peninsula. We detected heterozygote individuals of the 28S rDNA gene in Moldavia, Ukraine and in the southern part of Turkey. These suggest that the two clades can hybridise. Furthermore, the presence of European type homozygote individuals has been confirmed in the southern part of Turkey as well.

**Conclusions:**

We have shown that both post-glacial recolonization and recent expansion events influenced the present genetic structure of *P. platani*. The genetic patterns revealed at least two refugia during the last ice age: one in the Balkan Peninsula and the other in the Caucasus region. Recent expansion was detected in some European and Central Asian populations. The two main clades (Europe/Asia) show definite genetic differences; however, several hybrid individuals were found in the overlapping zone as well (stretching over Eastern Europe and the Anatolian Peninsula). Discrepancies in mitochondrial and nuclear data indicate introgressions in the southern part of the Anatolian Peninsula.

**Electronic supplementary material:**

The online version of this article (10.1186/s12862-018-1240-z) contains supplementary material, which is available to authorized users.

## Background

Biological invasions have been considered a major economic and conservation problem recently [1, 2]. The plane leaf miner, *Phyllonorycter platani* (STAUDINGER, 1870), is one of the most important invasive Gracillariidae species [3, 4]. Its colonisation history is well documented across Europe and its range expansion started in the second half of the nineteenth century [3, 5]. The colonisation process includes several jumps from the native origin (SE-Europe) to the Northern and North-Western Europe [5]. The dispersal occurred in anemochoral and antropochoral ways with passive transportation of mined leaves and/or saplings [5].

According to the Global Taxonomic Database of Gracillariidae [6] *Phyllonorycter platani* (STAUDINGER, 1870) has a large distribution area across Europe, the Anatolian Peninsula, Near-East and Central Asia. Šefrová [3, 5] suggests that it is native to Southern Europe and to Central Asia where the host plant (*Platanus orientalis*) is native. Lopez-Vaamonde et al. [4] considered the area of origin of *P. platani* as unknown.

Heinrich [7] described *Phyllonorycter felinella* HEINRICH, 1920 in California (US), but Deschka [8] synonymized this taxon with *Lithocolletis platani* STAUDINGER, 1870. The actual situation of the taxon was, until recently, uncertain. [8–11], [7, 12–14].

Leaf mining is a special kind of phytophagy where monophagous species are dominant. Host shifts are rare in the genus *Phyllonorycter* [15] and the plane leaf miner is considered oligophagous. Confirmed host plants are various plane trees (taxon names are according to the Catalogue of Life [16]): *Platanus hybrida* BROT., *P. occidentalis* L., *P. orientalis* L. and *P. racemosa* NUTTAL [11]. However, mines have been observed mainly on *P. orientalis* (Austria, Bulgaria, Canary Islands, France, Greece, Italy, Macedonia, Romania, Spain, Tajikistan, Turkey, Turkmenistan, and UK) and *P. hybrida* (Austria, Croatia, Denmark, France, Italy, Poland, Portugal, Slovakia and UK) (De Prins personal communication)*.*

*Platanus orientalis* is native to the eastern part of the Mediterranean, across the Anatolian Peninsula and the Caucasus to Central Asia, especially in the coastal areas and river valleys [17–20]. The precise determination of native area boundaries is not possible anymore because the species was planted in ancient times [18, 20–23]. Plane trees are the most common ornamental and alley trees in temperate, Mediterranean and subtropical cities [24]. Therefore, aesthetic damage or early defoliation caused by *P. platani* is of high significance [7, 25, 26].

Most genetic analyses of *Phyllonorycter* species represent taxonomic works and use the mtDNA barcode fragment [27–29]. Others studied leaf miners and their host plant relationship using 28S rDNA marker [15, 30]. However, only a few studies used molecular data to reveal the colonisation history of *Phyllonorycter* [31, 32] or other Gracillariid leaf miner species [33].

Only a limited number of sequences were available in the various databases (e.g. NCBI, BOLD) for *P. platani* before this study. They include phylogenetic works of *Phyllonorycter* species [15, 30], outgroup data for *Cameraria* microsatellite markers [34], and parasitoid gut content [35]. In one of our previous works, 31 individuals were sequenced for the 520 bp long fragment of the 3′ end of the COI gene, which represented only 4 haplotypes for Europe [36].

Our aims were (i) to assess the current phylogeographic pattern of this species. (ii) To determine the origin of *P. platani* and its possible refugial areas. (iii) To reveal factors influencing the recent genetic pattern, especially the isolation by distance and/or geographic isolation of certain populations.

## Materials and methods

### Sampling and molecular methods

**Fig. 1:**
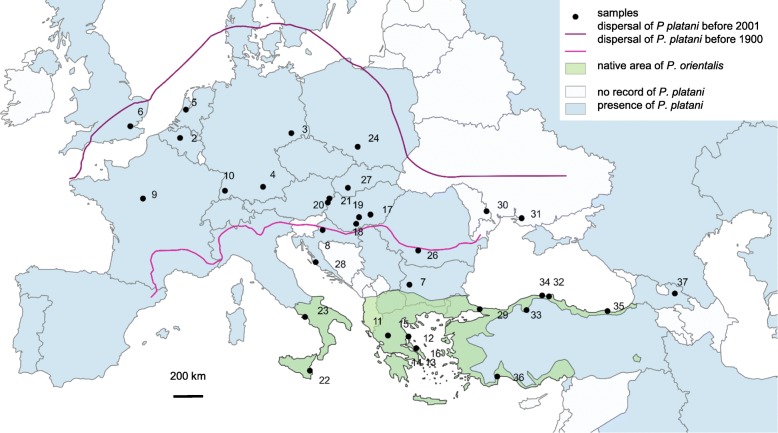
The native range of *Platanus orientalis* L.; current and previous distribution of *Phyllonorycter platani* (STAUDINGER 1870) and the sample locations in Europe. The native range of *Platanus orientalis* L. according to Feng et al. [3, 5]; current distribution of *Phyllonorycter platani* (STAUDINGER 1870) according to Global Taxonomic Database of Gracillariidae (www.gracillariidae.net); previous distribution as stated by Šefrová [3, 5]

We collected larvae and pupae from 38 populations of *P. platani*, two population of *P. issikii*, and one of *P. maestingella* (Table 1, Fig. 1). The identification of the species was based on damage symptoms (type and locality of the mine) and the host plants. All samples were stored in 96% ethanol at 4 °C until DNA extraction. Voucher specimens and extracted DNA samples are stored at the institute’s collection.

**Table 1:** Origin, number of haplotypes/alleles per genetic markers and the host plants of investigated *Phyllonorycter* species

Species	ID	Region	Country	Location	Lat.	Long.	COI		28S		Host plant
							n	No.	n	No.	
*P. platani*	1	North America	USA	Monterey	36.60	− 121.90	11	4	10	1	*Platanus sp.*
	2	N-NW Europe	Belgium	Brussels	50.72	5.62	6	1	1	1	*Platanus sp.*
	3	N-NW Europe	Germany	Dresden	51.05	13.74	10	1			*Platanus sp.*
	4	N-NW Europe	Germany	Freising	48.40	11.74	10	1	3	1	*Platanus sp.*
	5	N-NW Europe	Netherlands	Rotterdam	51.92	4.48	4	1	1	1	*Platanus sp.*
	6	N-NW Europe	United Kingdom	London	51.51	−0.16	10	1			*Platanus sp.*
	7	S-C Europe	Bulgaria	Sofia	42.71	23.33	10	1			*Platanus sp.*
	8	S-C Europe	Croatia	Zagreb	45.81	15.97	9	2			*Platanus sp.*
	9	S-C Europe	France	Suèvres	47.67	1.46	10	1			*Platanus sp.*
	10	S-C Europe	Germany	Freiburg	48.00	7.85	9	2	1	1	*Platanus sp.*
	11	S-C Europe	Greece	Kastraki	39.72	21.62	10	4	10	1	*Platanus sp.*
	12	S-C Europe	Greece	Paralia Chiliadou	38.67	23.93	6	2	1	1	*Platanus orientalis*
	13	S-C Europe	Greece	Kathenoi	38.57	23.77	5	3	1	1	*Platanus orientalis*
	14	S-C Europe	Greece	Stropones	38.62	23.89	4	1			*Platanus orientalis*
	15	S-C Europe	Greece	Milopotamos	39.38	23.20	3	2	2	1	*Platanus orientalis*
	16	S-C Europe	Greece	Steni Dirfios	38.58	23.84	4	1			*Platanus orientalis*
	17	S-C Europe	Hungary	Csongrád	46.71	20.15	2	1	1	1	*Platanus sp.*
	18	S-C Europe	Hungary	Dávod	46.00	18.92	2	1	1	1	*Platanus sp.*
	19	S-C Europe	Hungary	Hajós	46.4	19.11	2	2			*Platanus sp.*
	20	S-C Europe	Hungary	Kőszeg	47.39	16.54	3	1			*Platanus sp.*
	21	S-C Europe	Hungary	Sopron	47.68	16.58	6	2			*Platanus acerifolia*
	22	S-C Europe	Italy	Pantalica	37.13	14.98	10	2	2	1	*Platanus sp.*
	23	S-C Europe	Italy	Pompei	40.75	14.5	10	1			*Platanus sp.*
	24	S-C Europe	Poland	Katowice	50.26	19.03	9	3	3	1	*Platanus sp.*
	26	S-C Europe	Romania	Craiova	44.32	23.79	9	1			*Platanus sp.*
	27	S-C Europe	Slovakia	Nitra	48.31	18.09	9	3	1	1	*Platanus sp.*
	28	S-C Europe	Croatia	Zadar	44.13	15.24	6	2			*Platanus sp.*
	29	S-C Europe	Turkey	Istanbul	41.04	28.99	9	2	5	1	*Platanus acerifolia*
	30	E Europe	Moldova	Tiraspol	46.84	29.63	10	2	9	2	*Platanus sp.*
	31	E Europe	Ukraine	Tsiurupynsk	46.60	32.72	9	2	8	2	*Platanus orientalis*
	32	N Anatolia	Turkey	Ayancik	41.90	34.58	8	4	5	1	*Platanus sp.*
	33	N Anatolia	Turkey	Karabük	41.21	32.62	9	2	3	1	*Platanus sp.*
	34	N Anatolia	Turkey	Catalzeytin	41.95	34.21	8	1	2	1	*Platanus sp.*
	35	N Anatolia	Turkey	Trabzon	41.00	39.72	9	4	1	1	*Platanus sp.*
	36	S Anatolia	Turkey	Antalya	36.88	30.71	9	1	9	2	*Platanus sp.*
	37	Caucasus	Georgia	Telavi	41.92	45.48	10	3	10	1	*Platanus sp.*
	38	Central Asia	Uzbekistan	Samarkand	39.65	66.96	10	1	10	1	*Platanus sp.*
	39	Central Asia	Kyrgyzstan	Bishkek	42.88	74.6	4	1	3	1	*Platanus sp.*
*P. issikii*		S-C Europe	Hungary	Sopron	47.68	16.58	2	1			*Tilia sp.*
		S-C Europe	Hungary	Kőszeg	47.39	16.54	1	1			*Tilia sp.*
*P. maestingella*		S-C Europe	Hungary	Sopron	47.68	16.58	1	1			*Fagus sylvatica*

*n* number of individuals, *No.* number of haplotypes/alleles, *N-NW Europe* North-Northwest Europe, *S-C Europe* South and Central Europe, *E Europe* East Europe, *N Anatolia* northern Part of Anatolia, *S Anatolia* southern part of Anatolia

DNA was extracted from entire bodies using: a) GenElute Mammalian Genomic DNA Miniprep Kit (Sigma-Aldrich), b) E.Z.N.A.® Tissue DNA Kit and c) AquaGenomic Kit following the manufacturer’s protocol. Eluted DNA was stored at − 20 °C.

A 1242 bp-long region of the COI gene was amplified for 284 individuals by using Pat, (5’-TCCAA TGCACTAATCTGCCATATTA-3′) and Lep2F (5′- ATTCAACAATCATAAAGATATTGG-3′) primers [37, 38], as well as two additional primers Dick (5’-CCAACAGGAATTAAAATTTTTAGATGA-3′) [38] and Pc6F (5’-GCCCCAGATATAGCATTTCC-3′) [39]. PCR conditions included an initial denaturation step at 94 °C for 2 min, followed by 34 cycles at 94 °C for 30 s, 47 °C for 1 min and 72 °C for 1 min 30 s with a final extension step that lasted 10 min at 72 °C.

We amplified an 893 bp fragment of the 28S rDNA for 103 individuals from five selected populations (Table 1) using D1F (5’-ACCCGCTGAATTTAAGCATAT-3′) and D3R (5’-TAGTTCACCATCTTTCGGGTC-3′) primers [40]. We used the polymerase chain reaction as described by Lopez-Vaamonde et al. [40].

Sequences were generated (bidirectionally) at the Eurofin’s Laboratory (Ebersberg, Germany). Sequences are available via GenBank with accession numbers KY952988–KY953017.

### Data analysis

For nuclear DNA (28S) analyses, 103 individuals were used and 284 individuals were used for mitochondrial DNA (COI) analyses (Table 1). Every specimen used for 28S rDNA analyses was amplified for COI too. Sequences were visualized using Sequence Scanner and then aligned using ClustalX [41]. After haplotypes were identified, those represented by only a single individual were verified by additional sequencing of an independent amplicon. *P. issikii* and *P. maestingella* were used as outgroups. Genetic distances were calculated with MEGA 5.02 [42].

### Phylogenetic analyses

Maximum likelihood (ML) analysis was performed under GTR + I model with MEGA 5.02. The level of support for individual nodes was evaluated by bootstrapping with 5000 replicates. We used jModeltest 2.1.2 [43, 44] to select the best model of nucleotide substitution with Akaike Information Criterion (AIC) [45].

### Population structure

Patterns of molecular diversity based on the mtDNA sequences between and within populations were assessed by estimating: nucleotide diversity (π) [46], transition/transversion ratio, haplotype diversity (h) [47, 48] using the software Arlequin version 3.5.1.2 [49].

### Demographical expansion

Population dynamics analyses were performed on different geographical scales (all dataset, between continents, within continents, with special emphasis of European populations). For the estimation of Tajima’s D statistics [50] and Fu’s Fs [51] Arlequin 3.5.1.2 was used with 10,000 permutations [49]. On the small sample size (< 30 individuals) we additionally used DnaSp 5.10 [52] to estimate R2 [53].

### Phylogeographical analysis

Spatial analysis of molecular variance (SAMOVA) was performed using SAMOVA v1.0 [54]. The program was run 1023 iterations. K values were tested, starting from two until the value for which F_CT_ reached a plateau [55].

In addition, alternative geographical groups were tested with Analysis of Molecular Variance (AMOVA) [56–58] with Arlequin 3.5.1.2 [49]. The statistical significance of variance components in AMOVA was tested with 1000 permutations.

Isolation by distance was evaluated using Mantel test [59] with MANTEL NON-PARAMETRIC CALCULATOR ver. 2.0 [60]. Natural algorithms of geographical linear distances (km) between localities were correlated with the respective Tamura-Nei genetic distances [61] and were calculated with MEGA v.5.02 [42] with 1000 random iterations to obtain statistical inferences at α = 1%.

Statistical parsimony network (SP) [62] was created using a TCS 1.2.1 [63]. The nesting design was constructed on the SP network [64, 65].

## Results

### COI mtDNA

**Fig. 2:**
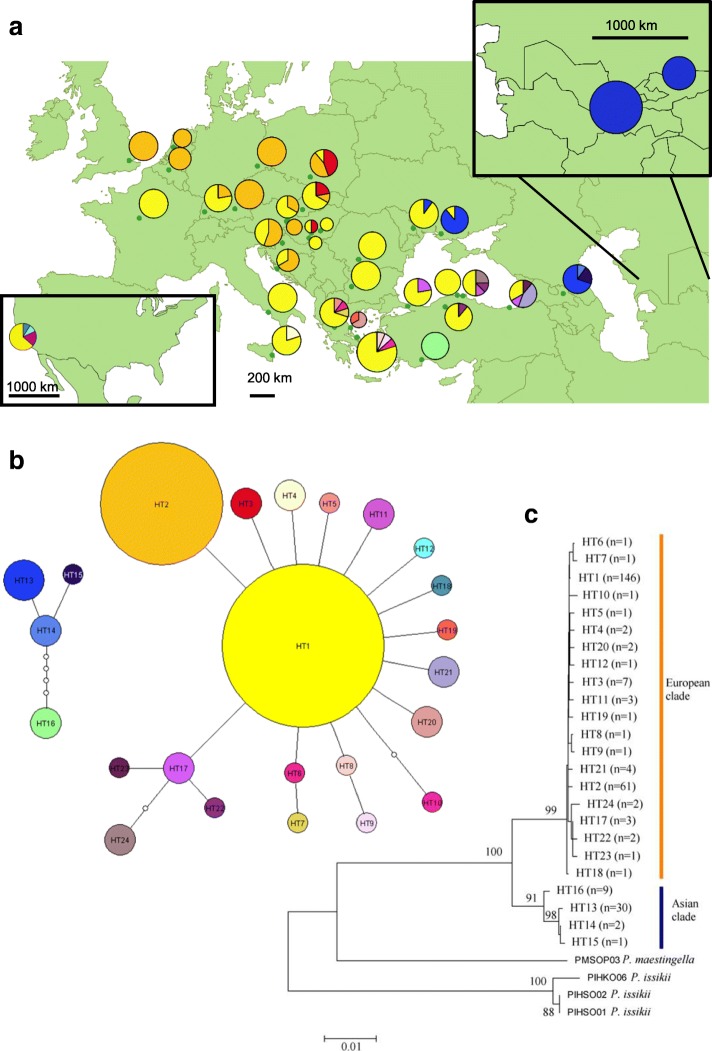
Distribution and phylogenetic relationship of *Phyllonorycter platani* mitochondrial haplotypes. **a**: Distribution of COI haplotypes in Europe; **b**: Statistical parsimony networks for all haplotypes (empty circles indicate missing or theoretical haplotypes); **c**: ML consensus tree of all COI haplotypes. Numbers above branches indicate ML probabilities (> 0.60)

Twenty-four haplotypes were detected on the 1242 bp long fragment of the COI gene (Table 1, Fig. 2, Additional file 1). The number of variable sites was 43 (3.5%). Approximately the half of these were located on the barcode part of the gene.

There were 22 mutational steps between the Asian and European clades. Pairwise genetic distances between Asian (HT13–16) and European (HT1–12, 17–24) or between Asian (HT13–16) and North American (HT1, 11–12, 18) haplotypes are lower (1.80–2.30%) than the interspecific distance among related *Phyllonorycter* species (8.52–10.68%).

Average sequence divergence between Asian and European clades (2.08%) was higher than the intrapopulation level (0.11%; 0.20%). Divergence data shows that the population from the southern part of Anatolia (HT16) is closer to the Caucasian and Central Asian group (0.46%) than to the European group (1.88%). The genetic divergence between European and North American haplotypes was rather low (0.1–0.4%) in comparison to the outgroups (8.4–11.0%). ML tree support two clades with 100% probability: 1) the Asian and 2) the European (including the North American haplotypes) (Fig. 2). The HT1 was detected in 51.4% of the individuals and it is the most common haplotype in Europe, the northern part of the Anatolian Peninsula, and North America (Fig. 2). HT2 was frequent (21.5% of the total 288 individuals) in Western, North-Western, and Central Europe; we also found it in some Southern European populations (Croatia). HT3 (2.5%) was detected only in Central Europe. HT13 (10.6%) was detected from the Caucasus (Georgia), Eastern Europe (Moldavia, Ukraine) and it was common in the Central Asian populations (Uzbekistan, Kyrgyzstan). We detected the unique haplotype HT16 (3.6%) in the southern part of Anatolia. The Mediterranean part of Europe was represented by several unique haplotypes (HT4–10, HT19, HT20) similar to the northern part of Anatolia (HT17, HT21–24). The HT11 was detected from the northern part of Anatolia and North America. The HT12 and HT18 revealed only from North America. The HT14–15 were unique haplotypes from Caucasus (Georgia).

We observed moderate values of the diversity indices in the species (h = 0.68, π = 0.55%) (Table 2). Haplotype diversities were moderate and nucleotide diversities were low in both the Asian (h = 0.49, π = 0.26%) and the European clade (North American samples included) (h = 0.58, π = 0.08%). Based on the high rate of the Caucasian (Georgia) diversity indices (h = 0.51, π = 0.05%), and the homogeneity of Central Asian populations (Uzbekistan, Kyrgyzstan) a recent expansion to Central Asia from the Caucasus is assumed. The homogeneity of the population from the southern part of Anatolia (Antalya) suggests a founder effect. We observed high diversity indices (h = 0.60, π = 0.06%) for the North American population. We revealed 0.57 haplotype diversity and 0.08% nucleotide diversity in the European specimens.

**Table 2:** Summary of genetic diversity indices and neutrality tests for the COI gene

	n	S	No.	h ± SD	π (%) ± SD	Tajima’s D	Fu’s Fs	R2
European haplogroup	242	41	21	0.5780 ± 0.0291	0.0767 ± 0.0582	−2.470**	−17.852**	Ø
Asian haplogroup	42	27	5	0.4855 ± 0.0780	0.2644 ± 0.1541	−1.613	4.037	Ø
North America	11	3	4	0.6000 ± 0.1539	0.0556 ± 0.0513	−1.114	−1.525	0.156*
N-NW Europe	40	0	1	0.0000 ± 0.0000	0.0000 ± 0.0000	0.000	0.000	Ø
S-C Europe	191	40	19	0.4656 ± 0.0435	0.0709 ± 0.0552	−2.553**	−16.455**	Ø
East Europe	19	25	2	0.5263 ± 0.0400	1.0594 ± 0.5576	3.289	19.278	0.132*
N Anatolia	34	28	8	0.7487 ± 0.0579	0.7305 ± 0.3833	1.142	6.007	Ø
S Anatolia	9	0	1	0.0000 ± 0.0000	0.0000 ± 0.0000	0.000	0.000	n.r.
Caucasus	10	2	3	0.5111 ± 0.1643	0.0537 ± 0.0506	−0.184	−0.272	0.162*
Central Asia	14	0	1	0.0000 ± 0.0000	0.0000 ± 0.0000	0.000	0.000	n.r.
Total	284	24	24	0.6784 ± 0.0244	0.5516 ± 0.2881	−0.024	0.974	Ø

(*n*) number of individuals sampled, (*S*) number of polymorphic sites, (*No*) number of haplotypes, (*h*) haplotype diversity, (*π*) nucleotide diversity, with standard deviation (*SD*), (*D*) and (*Fs*) statistic for neutrality test, (*Ø*) too large sample size, (*n.r.*) not relevant, ** *p* < 0.01, * *p* < 0.05

Genetic population structure correlated with geographic distances (Mantel test). The correlation coefficient (r) indicates either a moderate (full dataset *r* = 0.361, Ρ = 0.005), or a weak correlation: European clade (including North American samples, *r* = 0.01, Ρ = 0.010); and European population only (without the Eastern European samples, *r* = 0.206, Ρ = 0.005).

As the F_CT_ values reached a plateau at K = 2 and single-population groups were formed when K > 2, we used two as the optimal number of population groups. The two groups found by the SAMOVA are geographically consistent and correspond to regions (Table 3). On the full dataset, the two main groups (the first group contains populations from the south part of Turkey, Ukraine, Georgia, Uzbekistan, and Kyrgyzstan while the second group contains all others) actually match to the two main clades. Most of the molecular variance is found among groups (Va = 94.32%, *p* < 0.001), but ca. 2.55% (Vb) of variance is still found among populations within groups (*p* < 0.001). We can detect only slight gene flow between the two main groups. In the second arrangement, when we used the European clade (without the East-European samples which constitute the hybrid zone), the first group contains Northern and North-Western European populations (Brussels, Dresden, Freising, London, Rotterdam), and the second one contains Southern and Central European populations with the North American samples (Va = 51.57%; Vb = 40.64%). In the third arrangement we used only the European samples (without Eastern European and North American samples) and received similar results (Va = 51.23%, Vb = 40.71%).

**Table 3:** Analysis of molecular variance based on the two groups defined by SAMOVA. (***p < 0.001)

Groups	Source of variation		var %	Fixation indices	
Asian haplogroup	Among groups	Va	94.32	F_CT_=	0.94316***
European haplogroup	Among populations within groups	Vb	2.55	F_SC_=	0.4493***
	Within populations	Vc	3.13	F_ST_=	0.9687***
N-NW Europe	Among groups	Va	51.57	F_CT_=	0.51575***
S-C Europe + N America	Among populations within groups	Vb	7.78	F_SC_=	0.1607***
	Within populations	Vc	40.64	F_ST_=	0.59357***
N-NW Europe	Among groups	Va	51.23	F_CT_=	0.51234***
S-C Europe	Among populations within groups	Vb	8.05	F_SC_=	0.16516***
	Within populations	Vc	40.71	F_ST_=	0.59288***

Most of the Tajima’s D and Fu’s Fs indices are not significant (Table 2); we detect significant negative values only from the European clade (D = − 2.470, Fs = − 17.852), and from the Southern and Central European subgroup (D = − 2.553, Fs = − 16.455), which suggests recent population expansion. On the other hand, the R2 indices, which better fit small sample sizes, are significant (*p* = 0.00) in the populations from North America (R2 = 0.156), Caucasus (R2 = 0.162), and Eastern Europe (R2 = 0.132).

### 28S rDNA

**Fig. 3:**
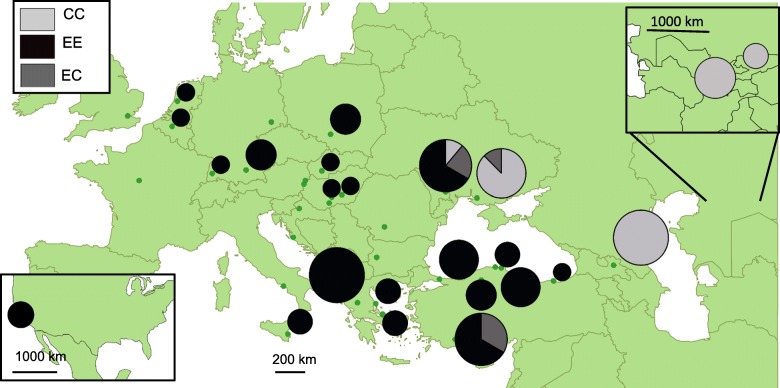
Distribution of *Phyllonorycter platani* genomic (28S) alleles in Europe and Asia

Two alleles were identified on the 893 bp long fragment of the 103 specimens sequenced from 11 selected populations (Fig. 3). We revealed 0.10% divergence between the two alleles, which means one variable site (T/G transversion). These two alleles represent the two main clades. Populations from N-NW Europe, S-C Europe, N Anatolia, and North America were homogenous and contain the European allele only. The population from the Central Asia (Kyrgyzstan, Uzbekistan) and the Caucasus (Georgia) contains the Asian allele only while samples from Eastern Europe (Moldova, Ukraine) and from the southern part of Anatolia were represented by homo and heterozygote individuals as well (Table 4, Fig. 3).

**Table 4:** COI haplotypes and 28S rDNA alleles of *P. platani* individuals

Region	Country	Location	COI HT	28S allele		
				EE	EC	CC
E Europe	Moldova	Trisapol	HT1	6	1	1
			HT13		1	
	Ukraine	Tsiurupynsk	HT1			1
			HT13		1	6
				6	3	8
N America	USA	Monterey	HT1	6		
			HT11	2		
			HT12	1		
			HT18	1		
				10	–	–
S Anatolia	Turkey	Antalya	HT16	6	3	
				6	3	–
N Anatolia	Turkey	Ayancik	HT1	3		
			HT22	1		
			HT24	1		
		Karabük	HT1	3		
		Catalzeytin	HT1	2		
		Trabzon	HT23	1		
				11	–	–
S-C Europe	Turkey	Istambul	HT1	4		
			HT17	1		
	Greece	Kastraki	HT1	7		
			HT5	1		
			HT6	1		
			HT7	1		
		Paralia Chiliadou	HT8	1		
		Kathenoi	HT9	1		
		Milopotamos	HT19	1		
			HT20	1		
	Italy	Pantalica	HT4	2		
	Hungary	Csongrád	HT1	1		
		Dávod	HT1	1		
	Poland	Katowice	HT2	1		
			HT3	2		
	Slovakia	Nitra	HT3	1		
				27	–	–
N-NW Europe	Belgium	Brussels	HT2	1		
	Netherlands	Rotterdam	HT2	1		
	Germany	Freising	HT2	3		
		Freiburg	HT1	1		
				6	–	–
Caucasus	Georgia	Telavi	HT13			7
			HT14			2
			HT15			1
				–	–	10
Central Asia	Uzbekistan	Samarkand	HT13			10
	Kyrgyzstan	Bishkek	HT13			3
				–	–	13
Altogether				66	6	31

## Discussion

### Inter and intraspecific diversity

The interspecific divergence on the COI gene is high in the Lepidopteran families [66–69]. In the *Phyllonorycter* genus especially, an extremely high interspecific divergence was observed (19.60% between *P. obandai* and *P. salictella*) [28]. In the *Phyllonorycter ulicicolella* species group, Laštůvka et al. [29] noted a minimum interspecific pairwise genetic distance of 3.1%. The average divergence between the *Phyllonorycter* species is 6.04% on the barcode fragment [31].

A wide range of varying intraspecific divergence exists for the different Lepidoptera species: e.g. 0.00–0.50% within *Macrosaccus* species, 0.49% within *Cameraria ohridella*, 0.17–2.07% *Choristoneura sp*., 0.11–3.22% *Hyles sp.*, 1.30–2.50% *Sciadia tenebraria,* 1.50–4.10% *Epirrita autumnata*, 1.93% within *P. salictella* [28, 33, 66, 68–70]. Average intraspecific divergences are 0.94% among *Phyllonorycter* species [31]. The genetic distances values (0.10–2.30%) between *P. platani* haplotypes are similarly high to another plane feeding Lepidopteran species of *Acalyptris platani* (1.80–2.30%) [71]. In the case of *P. issikii*, the average distance (5.13 ± 0.003%) between the two clades is approximately two times higher [32] than between our two major clades of *P. platani* (2.08 ± 0.369%). The maximum intraspecific divergence is 2.96% for *P. issikii* and 1.38% for the putative new species [32], while we revealed 0.39% for the Asian and 0.25% for the European clade of *P. platani*.

The interspecific genetic divergence on 28S rDNA varies 0.20–25.00%, while the average intraspecific divergence is lower than 0.50% in the genus *Phyllonorycter* [30]. We detect much lower divergence between Asian and European alleles (0.10%). This was represented by a single variable position (T/G transversion). In comparison, *P. issikii* revealed 7 diagnostic substitution differences on this fragment of the 28S rDNA [32]. The high intraspecific divergence on mtDNA with low nuclear divergence does not support the presence of a cryptic species in our case [68]. In addition, we revealed heterozygote individuals from three populations for 28S rDNA, which shows that the individuals from the two clades can hybridize.

The haplotype diversity (h) of the plane leaf miner (0.68) compared to other Lepidopteran species (*Aglais urticae* 0.96) is medium-high [72]. The nucleotide diversity (π) of the full dataset (0.55%) is also medium-high (*Dioryctria* species 0.03–0.35%, *Hyles* genus 0.03–2.71%, *Papilio* species 0.26–2.71%) [33, 66, 72]. On the other hand, the 0.08% nucleotide diversity of the European samples compared to another leaf miner Lepidoptera, *Cameraria ohridella* (π = 0.17% in the natural area; and π = 0.09% in the other European places) is lower [33].

### Phylogeographic pattern

Past and recent gene flow events determine the geographical pattern of populations [73]. One of them can be the influence of ice ages followed by species recolonization [74]. The other can be recent expansion or invasion. The latter is well described for the *P. 12platani* populations [3, 5, 75]; however, possible glacial refugial areas of the species remain unclear [4]. In our study, we demonstrated that both post-glacial recolonization and recent expansion events influenced the present genetic structure of *P. platani*.

According to the coalescent theory, the most frequent haplotype is supposed to be the most ancient one [76]. However, some authors [77] infirm this for Lepidopteran species. In our case these the modal haplotypes are HT1 (51.4%), HT2 (21.5%), HT13 (10.6%), HT3 (2.5%) (Fig. 2). The analysis of the population dynamics (Table 2, Tajima’s D, Fu’s Fs) and the geographical distribution pattern (Fig. 2) of both HT2 and HT3 suggest that these are rare haplotypes at the edge of the original distribution area of the species and that these haplotypes have gone to fixation under the range expansion and occurred more frequently in the recently colonized area [73, 78]. The diversity indices (Table 2) of the population from the southern part of the Anatolian Peninsula also show a possible recent expansion effect. Based on our results, there were likely two glacial refugial areas during the last ice age: one in the Balkan Peninsula and the other in the Caucasus. Analysis of further populations from this region, especially from the Caucasus and the south coast of the Caspian Sea, could provide a better resolution of the geographic patterns and the intermediate haplotypes between the two clades.

All methods used for the statistical analyses (ML, divergence data, SAMOVA) support the existence of two main clades (European and Asian) and the further differentiation of the Asian clade. The genetic divergence between the European and the Asian clade is high (2.08%), but this is typical for the members of the family Gracillariidae [31, 69]. Haplotype diversity is moderate (h = 0.49) and nucleotide diversity is low (π = 0.26%) for the Asian clade. Values for the European clade (h = 0.58, π = 0.08%) show only moderate difference. Rapid expansion after bottlenecks causes similar diversity patterns [33, 79, 80]. We surmise that the last glacial period caused this bottleneck. Several studies deal with the effects of the ice ages on diversity and effective population size [74, 80–82]. Plane trees and their herbivore communities may have survived in only a few refuges in Southern Europe, the coastal part of the Anatolian Peninsula, the east coast of the Black Sea, and the south coast of the Caspian Sea [20, 83–86]. The Mediterranean refugial area was fragmented consisting of several small, dispersed areas with warm and relative humid microclimates such as rivers floodplains, 400–800 m elevations, seaside, deep valleys etc. [83, 84, 86]. Médail and Diadema [84] describe 52 Mediterranean plant refugias in Europe. This may be the major reason for the high variability we found in the population from the Caucasus. Furthermore, it may also be the reason why only some of the Mediterranean populations were represented with high variations and why we found homogenous populations from the intermediate locations.

The HT16 (which is represented in the southern part of Anatolia) is linked more closely to the Asian haplotypes (HT13–15) than to the European. This suggests that there may have been a connection among the refuges of plane leaf miner populations during the interglacial periods.

Mantel test results and SAMOVA also support the view that the species survived the ice ages in several refugia because the isolation by distance values are moderate (*r* = 0.3605), and the variability value is high (Va = 94.32%) between the main two major clade.

### Recent expansion

The Asian clade is well differentiated, so we analysed the dynamics of the subgroups: the Caucasus, Central Asia and the southern part of Anatolia (Antalya). The population from Antalya has a homogenous haplotype pattern, which refers to a founder effect. Several little plant refugia are described from the Mediterranean Basin [84], but the refugia of the Anatolian Peninsula have not been located exactly in the surroundings of Antalya. Climate simulations predicted possible refugia mainly from the northern part of Anatolia for the warm summer-green trees such as the host plant [83]. The homogeneity of the populations from Central Asia (Uzbekistan, Kirghizistan) and the high diversity values (h = 0.5111, π = 0.0536%) of the Caucasian (Georgia) population suggest that *P. platani* may spread from the Caucasus to Central Asia recently.

The star-like shape of the SP network (Fig. 2) refers to recent demographical expansion from low effective population size [33, 73]. NCPA results are in accordance with Šefrová’s [3, 5] results; Šefrová stated that the plane leaf miner spread with jumps through Europe.

The populations of *P. platani* in Europe and in the north of the Anatolian Peninsula may have gone through a rapid range expansion after bottleneck (neutrality testes D = − 2.459, Fs = − 14.403). Populations from the France-Germany borderline, the eastern Alps and the eastern border of Germany compose the edges of the “W-NW” population supported by SAMOVA (“W-NW” and “S-C”), which are common barriers within Europe [85]. The low diversity values (h = 0.00, π = 0.00%) of “W-NW” group are consequences of the founder effect [79]. In the case of the “S-C” group, high haplotype diversities (0.466) with low nucleotide diversities (0.071%) resulted from a rapid expansion from small effective population size [79, 87]. Presumably, the HT2 and the HT3 were rare mutations that evolved on the edges of the original area and, after population expansion, they became fixed in the new populations [73]. The outcome of neutrality tests (R2 = 0.156) – similar to the diversity indices – suggests that sudden demographic expansion shaped the current pattern of intraspecific diversity of the North American population.

However, while analysing our dataset we have to take into consideration that three factors (1: population structure, 2: genetic diversity and 3: sampling scheme) might have major influence on the quantification of population size changes (see Chikhi et al. for further details [88]). In our case both the sampling scheme and the various genetic structure of the different populations may have an effect.

The results of the COI and 28S rDNA sequences show that the two main clades can hybridize. We found two possible hybrid zones. One of them is located in the eastern part of Europe; populations from Moldavia, Ukraine contain both of Asian and European haplotypes. In addition, we detected hybrid individuals from the Moldavian population with the nuclear marker. The other hybrid zone is located in the southern part of Anatolia. The detected unique COI haplotype (HT16) is more closely linked to the Asian haplotypes than to the European haplotypes. The revealed allelic pattern with the 28S rDNA marker shows the presence of heterozygotes and European-type homozygote individuals. This discrepancy with mitochondrial and nuclear data shows that there were introgressions in the southern part of Anatolia. In most cases, mito-nuclear discrepancies are the results of possible secondary contact zones after isolation [89, 90]. The extension of the hybrid zones is unknown at the moment because of the low number of sampled populations from these regions.

Our results also confirmed that the synonymization of *Lithocolletis felinella* HEINRICH, 1920 to *Phyllonorycter platani* (STAUDINGER, 1870) is required, however a morphotaxonomic approval is desirable.

## Conclusions

We have shown that both post-glacial recolonization and recent expansion events influenced the present genetic structure of *P. platani*. The genetic patterns revealed at least two refugia during the last ice age: one in the Balkan Peninsula and the other in the Caucasus region. Recent expansion was detected in some European and Central Asian populations. The two main clades (Europe/Asia) show definite genetic differences; however, several hybrid individuals were found in the overlapping zone as well (stretching over Eastern Europe and the Anatolian Peninsula). Discrepancies in mitochondrial and nuclear data indicate introgressions in the southern part of the Anatolian Peninsula.

## Additional file

Additional file 1: COI haplotype distribution of *Phyllonorycter platani*. Number of COI haplotypes (HT1–24) in the countries/locations investigated. (DOCX 32 kb)

## Abbreviations

ML: maximum likelihood
NCPA: nested clade analysis
SP: statistical parsimony
