# Supplementary material for: Phylogeographic pattern of the plane leaf miner, Phyllonorycter platani (STAUDINGER, 1870) (Lepidoptera: Gracillariidae) in Europe
Source: BMC Evol Biol. 2018 Sep 6;18:135. doi: 10.1186/s12862-018-1240-z (PMC6127947; doi:10.1186/s12862-018-1240-z)
Supplement: Supplementary file 1 — COI haplotype distribution of Phyllonorycter platani. Number of COI haplotypes (HT1–24) in the countries/locations investigated. (DOCX 32 kb) [file 12862_2018_1240_MOESM1_ESM.docx]

**Additional file 1**

COI haplotype distribution of *Phyllonorycter platani*

| **Country** | **Location** | **HT1** | **HT2** | **HT3** | **HT4** | **HT5** | **HT6** | **HT7** | **HT8** | **HT9** | **HT10** | **HT11** | **HT12** | **HT13** | **HT14** | **HT15** | **HT16** | **HT17** | **HT18** | **HT19** | **HT20** | **HT21** | **HT22** | **HT23** | **HT24** | **n** |
| --- | --- | --- | --- | --- | --- | --- | --- | --- | --- | --- | --- | --- | --- | --- | --- | --- | --- | --- | --- | --- | --- | --- | --- | --- | --- | --- |
| USA | Monterey | 7 |  |  |  |  |  |  |  |  |  | 2 | 1 |  |  |  |  |  | 1 |  |  |  |  |  |  | **11** |
| Belgium | Brussel |  | 6 |  |  |  |  |  |  |  |  |  |  |  |  |  |  |  |  |  |  |  |  |  |  | **6** |
| Germany | Dresden |  | 10 |  |  |  |  |  |  |  |  |  |  |  |  |  |  |  |  |  |  |  |  |  |  | **10** |
| Germany | Freising |  | 10 |  |  |  |  |  |  |  |  |  |  |  |  |  |  |  |  |  |  |  |  |  |  | **10** |
| Netherlands | Rotterdam |  | 4 |  |  |  |  |  |  |  |  |  |  |  |  |  |  |  |  |  |  |  |  |  |  | **4** |
| United Kingdom | London |  | 10 |  |  |  |  |  |  |  |  |  |  |  |  |  |  |  |  |  |  |  |  |  |  | **10** |
| Bulgaria | Sofia | 10 |  |  |  |  |  |  |  |  |  |  |  |  |  |  |  |  |  |  |  |  |  |  |  | **10** |
| Croatia | Zagreb | 4 | 5 |  |  |  |  |  |  |  |  |  |  |  |  |  |  |  |  |  |  |  |  |  |  | **9** |
| France | Suèvres | 10 |  |  |  |  |  |  |  |  |  |  |  |  |  |  |  |  |  |  |  |  |  |  |  | **10** |
| Germany | Freiburg | 7 | 2 |  |  |  |  |  |  |  |  |  |  |  |  |  |  |  |  |  |  |  |  |  |  | **9** |
| Greece | Kastraki | 7 |  |  |  | 1 | 1 | 1 |  |  |  |  |  |  |  |  |  |  |  |  |  |  |  |  |  | **10** |
| Greece | Paralia Chiliadou | 5 |  |  |  |  |  |  | 1 |  |  |  |  |  |  |  |  |  |  |  |  |  |  |  |  | **6** |
| Greece | Kathenoi | 3 |  |  |  |  |  |  |  | 1 | 1 |  |  |  |  |  |  |  |  |  |  |  |  |  |  | **5** |
| Greece | Stropones | 4 |  |  |  |  |  |  |  |  |  |  |  |  |  |  |  |  |  |  |  |  |  |  |  | **4** |
| Greece | Milopotamos |  |  |  |  |  |  |  |  |  |  |  |  |  |  |  |  |  |  | 1 | 2 |  |  |  |  | **3** |
| Greece | Steni Dirfios | 4 |  |  |  |  |  |  |  |  |  |  |  |  |  |  |  |  |  |  |  |  |  |  |  | **4** |
| Hungary | Csongrád | 2 |  |  |  |  |  |  |  |  |  |  |  |  |  |  |  |  |  |  |  |  |  |  |  | **2** |
| Hungary | Dávod | 2 |  |  |  |  |  |  |  |  |  |  |  |  |  |  |  |  |  |  |  |  |  |  |  | **2** |
| Hungary | Hajós | 1 |  | 1 |  |  |  |  |  |  |  |  |  |  |  |  |  |  |  |  |  |  |  |  |  | **2** |
| Hungary | Kőszeg |  | 3 |  |  |  |  |  |  |  |  |  |  |  |  |  |  |  |  |  |  |  |  |  |  | **3** |
| Hungary | Sopron | 4 | 2 |  |  |  |  |  |  |  |  |  |  |  |  |  |  |  |  |  |  |  |  |  |  | **6** |
| Italy | Pantalica | 8 |  |  | 2 |  |  |  |  |  |  |  |  |  |  |  |  |  |  |  |  |  |  |  |  | **10** |
| Italy | Pompei | 10 |  |  |  |  |  |  |  |  |  |  |  |  |  |  |  |  |  |  |  |  |  |  |  | **10** |
| Poland | Katowice | 1 | 4 | 4 |  |  |  |  |  |  |  |  |  |  |  |  |  |  |  |  |  |  |  |  |  | **9** |
| Romania | Craiova | 9 |  |  |  |  |  |  |  |  |  |  |  |  |  |  |  |  |  |  |  |  |  |  |  | **9** |
| Slovakia | Nitra | 6 | 1 | 2 |  |  |  |  |  |  |  |  |  |  |  |  |  |  |  |  |  |  |  |  |  | **9** |
| Croatia | Zadar | 2 | 4 |  |  |  |  |  |  |  |  |  |  |  |  |  |  |  |  |  |  |  |  |  |  | **6** |
| Turkey | Istambul | 7 |  |  |  |  |  |  |  |  |  |  |  |  |  |  |  | 2 |  |  |  |  |  |  |  | **9** |
| Moldova | Tiraspol | 9 |  |  |  |  |  |  |  |  |  |  |  | 1 |  |  |  |  |  |  |  |  |  |  |  | **10** |
| Ukraine | Tsiurupynsk | 1 |  |  |  |  |  |  |  |  |  |  |  | 8 |  |  |  |  |  |  |  |  |  |  |  | **9** |
| Turkey | Ayancik | 4 |  |  |  |  |  |  |  |  |  | 1 |  |  |  |  |  |  |  |  |  |  | 1 |  | 2 | **8** |
| Turkey | Karabük | 8 |  |  |  |  |  |  |  |  |  |  |  |  |  |  |  |  |  |  |  |  | 1 |  |  | **9** |
| Turkey | Catalzeytin | 8 |  |  |  |  |  |  |  |  |  |  |  |  |  |  |  |  |  |  |  |  |  |  |  | **8** |
| Turkey | Trabzon | 3 |  |  |  |  |  |  |  |  |  |  |  |  |  |  |  | 1 |  |  |  | 4 |  | 1 |  | **9** |
| Turkey | Antalya |  |  |  |  |  |  |  |  |  |  |  |  |  |  |  | 9 |  |  |  |  |  |  |  |  | **9** |
| Georgia | Telavi |  |  |  |  |  |  |  |  |  |  |  |  | 7 | 2 | 1 |  |  |  |  |  |  |  |  |  | **10** |
| Uzbekistan | Samarkand |  |  |  |  |  |  |  |  |  |  |  |  | 10 |  |  |  |  |  |  |  |  |  |  |  | **10** |
| Kirghizistan | Bishkek |  |  |  |  |  |  |  |  |  |  |  |  | 4 |  |  |  |  |  |  |  |  |  |  |  | **4** |
| **Total** | | **146** | **61** | **7** | **2** | **1** | **1** | **1** | **1** | **1** | **1** | **3** | **1** | **30** | **2** | **1** | **9** | **3** | **1** | **1** | **2** | **4** | **2** | **1** | **2** | **284** |
